# Prevalence of common mental disorders and treatment receipt for people from ethnic minority backgrounds in England: repeated cross-sectional surveys of the general population in 2007 and 2014

**DOI:** 10.1192/bjp.2021.179

**Published:** 2022-09-01

**Authors:** Gargie Ahmad, Sally McManus, Claudia Cooper, Stephani L. Hatch, Jayati Das-Munshi

**Affiliations:** Department of Psychological Medicine, Institute of Psychiatry, Psychology & Neuroscience, King's College London, UK; Violence and Society Centre, City, University of London, and National Centre for Social Research, UK; Division of Psychiatry, Faculty of Brain Sciences, University College London, UK; ESRC Centre for Society and Mental Health, King's College London, UK

**Keywords:** Ethnicity, mental health inequalities, treatment inequalities, common mental disorders, social epidemiology

## Abstract

**Background:**

Concerns persist that some ethnic minority groups experience longstanding mental health inequalities in England. It is unclear if these have changed over time.

**Aims:**

To assess the prevalence of common mental disorders (CMDs) and treatment receipt by ethnicity, and changes over time, using data from the nationally representative probability sample in the Adult Psychiatric Morbidity Surveys.

**Method:**

We used survey data from 2007 (*n* = 7187) and 2014 (*n* = 7413). A Clinical Interview Schedule – Revised score of ≥12 indicated presence of a CMD. Treatment receipt included current anti-depressant use; any counselling or therapy; seeing a general practitioner about mental health; or seeing a community psychiatrist, psychologist or psychiatric nurse, in the past 12 months. Multivariable logistic regression assessed CMD prevalence and treatment receipt by ethnicity.

**Results:**

CMD prevalence was highest in the Black group; ethnic variation was explained by demographic and socioeconomic factors. After adjustment for these factors and CMDs, odds ratios for treatment receipt were lower for the Asian (0.62, 95% CI 0.39–1.00) and White Other (0.58, 95% CI 0.38–0.87) groups in 2014, compared with the White British group; for the Black group, this inequality appeared to be widening over time (2007 treatment receipt odds ratio 0.68, 95% CI 0.38–1.23; 2014 treatment receipt odds ratio 0.23, 95% CI 0.13–0.40; survey year interaction P < 0.0001).

**Conclusions:**

Treatment receipt was lower for all ethnic minority groups compared with the White British group, and lowest among Black people, for whom inequalities appear to be widening over time. Addressing socioeconomic inequality could reduce ethnic inequalities in mental health problems, but this does not explain pronounced treatment inequalities.

Over the past two decades, tackling persisting inequalities in the experience of mental health problems and access to mental healthcare has become a public health priority in the UK. However, evidence on the extent of these issues remains limited for people from ethnic minority and migrant backgrounds, who remain more likely to face adverse or coercive pathways into mental healthcare, and poorer outcomes from psychological services.^1,2^ This long-standing issue was recently publicised with the review of the Mental Health Act in 2018, which noted particular concerns for people from Black Caribbean and Black African groups, who were most likely to be detained involuntarily.^3^ The most recent comprehensive surveys focusing on ethnicity and mental health were conducted nearly two decades ago.^4^ Changes in migration patterns and demographic shifts have occurred since, so the current population diversity is not reflected in research. Surveys either have not assessed mental health and treatment, have had too few participants from ethnic minority backgrounds, or lack sufficient granularity of ethnicity data, which compromises the ability of robust conclusions to be drawn to inform policies to address the issues.^5^

Previously in England, the nationally representative Adult Psychiatric Morbidity Survey (APMS) in 2007 found that, after age standardisation, ethnic differences were not found in men, but common mental disorder (CMD) rates were higher in South Asian women compared with White women overall (grouping White British and White Other categories together).^6^ CMDs include depression, anxiety, panic disorder, obsessive–compulsive disorder, post-traumatic stress disorder and social anxiety disorders, which are generally treated in primary care services.^7^ Initial analysis of 2014 APMS data found that CMDs were more common in Black and Black British women, and less common in White Other women. Treatments for CMD include antidepressant medication prescribed by a general practitioner (GP), talking therapy either on referral from GP or self-referral, or referral to community mental health teams if problems are more severe. Age, gender and ethnicity were the most pronounced factors related to treatment inequalities among people with poor mental health; notably, people from an ethnic minority background were less likely to be in treatment, with rates lowest in the Black group.^8,9^

## Study aims

To address this major gap in research, we used data from the latest two waves (2007 and 2014) of the nationally representative APMS, to assess the prevalence of CMDs and treatment receipt for CMDs, in people from ethnic minority backgrounds relative to White British people. In particular, we assessed whether these have changed over time. We hypothesised that there would be higher CMD prevalence in people from ethnic minority backgrounds, that mental health treatment receipt would be lower in people from ethnic minority backgrounds compared with White British people, and that these associations would be attenuated by adjustment for socioeconomic circumstances.

## Method

### Participants and setting

APMS interviewed people aged ≥16 years in England about their mental health, health service use, demographic characteristics and socioeconomic circumstances. A stratified, multi-stage probability sampling strategy was applied to obtain a sample nationally representative of adults in private households, comprising 7403 people in 2007 and 7546 people in 2014. Consistent questions were answered across both waves. Details on the survey and sampling have been published elsewhere.^10^

### Measures

#### Ethnicity

Both APMS waves used Office for National Statistics harmonised questions on self-identified ethnic group or background, which contain up to 18 subcategories. Collapsed categories were used as the full breakdown was not available in the archived data-set. The five broad ethnic groups analysed here are White British; White Other; Black (including Black African, Black Caribbean and Black British); Asian (including Asian British, Bangladeshi, Indian and Pakistani); and Mixed, Multiple or Other Ethnic Group.

#### Demographic and socioeconomic factors

Harmonised questions established age, gender, marital status (single, married or cohabiting, separated or divorced), educational qualifications (degree, teaching/nursing/higher national diploma, A Level, GCSE/equivalent, foreign/other or no qualifications), home tenure (owner-occupier, social renter or private renter (including other types of occupancy, such as home provided with the job)) and the National Statistics Socio-economic Classification (NS-SEC) for categories of occupational social class (managerial or professional occupations, intermediate occupations, small employers or own account workers, lower supervisory or technical occupations, semi-routine or routine occupations, or either never worked or not worked in the past year).

#### CMDs

CMDs were assessed with the Clinical Interview Schedule – Revised (CIS-R), a structured questionnaire administered by lay interviewers that measures experience of 14 different symptoms in the preceding month, with emphasis on the past week.^11^ Scores of ≥12 are indicative of a clinically significant disorder. The CIS-R has been validated across different cultural settings and can be matched to ICD diagnoses for comparative analysis purposes.^12^

#### Mental health treatment receipt

A variable was derived to indicate receipt of any of the following treatments: currently receiving antidepressant medication; currently receiving any counselling or therapy for mental, nervous or emotional problems; seeing a GP for a mental, nervous or emotional complaint in the past 12 months; or seeing a community psychiatrist, psychologist or psychiatric nurse in the past 12 months. Largely the same questions were asked in both 2007 and 2014 surveys (except that data for 2014 groups psychiatric nurse and intellectual disability nurse consultations together).

### Data analysis

All analysis was conducted in Stata version 15 for Windows.^13^ Samples from both APMS waves were combined in this analysis: from a total sample of 14 949, a complete-case sample of 14 600 was derived (2007, *n* = 7187; 2014, *n* = 7413), comprising those providing responses across all measures listed above, excluding any cases who had data missing for any measure we investigated. Survey weights were applied to account for household selection probability and item non-response, to ensure a nationally representative household population sample; a specific weight for analysis of the 2007 and 2014 combined sample was applied, using the Stata version 15 *svy* command (further details can be found in the APMS documentation).8

Our main exposure was ethnicity, and main outcomes were CMD prevalence and treatment receipt, with putative confounders being survey year and demographic and socioeconomic factors of age, gender, marital status, educational status, home tenure and NS-SEC occupational social class.

Bivariable analysis was used to describe differences in the distribution of mental health outcomes and confounders by ethnicity. Pearson's *χ*^2^-tests were used to explore associations between ethnicity and outcomes (each type of treatment was described separately here). Multivariable logistic regression was used to assess differences in CMD prevalence and treatment receipt by ethnicity, unadjusted and adjusted to investigate whether controlling for demographic and socioeconomic factors explained differences in these outcomes (the adjusted model for treatment receipt also adjusted for CMD prevalence). The first stage of adjustment controlled for age, gender and survey year. The second stage of adjustment additionally controlled for other confounders. Survey year was included as an interaction term across all models, and the adjusted Wald test was applied to test for effect modification. Where there was evidence of interaction, estimates were adjusted for survey year.

### Ethics

This study was performed in accordance with the Declaration of Helsinki, and written consent was obtained from all survey participants; details are contained in APMS documentation.^10^ The project was approved by the King's College London Psychiatry, Nursing and Midwifery Research Ethics Panel in 2019, under reference number LRS-18/19-10496.

## Results

### Demographic and socioeconomic profile

**Fig. 1:**
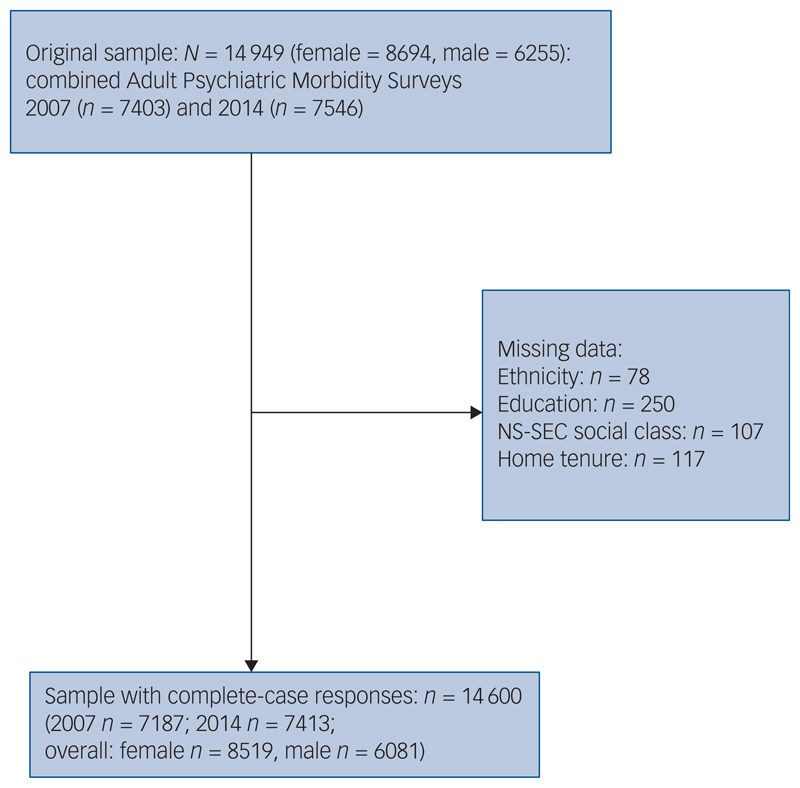
Sample flow diagram for 2007 and 2014 Adult Psychiatric Morbidity Survey data. NS-SEC, National Statistics Socio-economic Classification.

Sample characteristics of the combined 2007–2014 sample are provided in Table 1 (Supplementary Tables 1 and 2 available at https://doi.org/10.1192/bjp.2021.179 describe these characteristics separately for the 2007 and 2014 surveys, respectively). Fig. 1 illustrates how the complete-case sample was derived.

At least two-thirds of people from all ethnic minority groups were aged <45 years, where most of the White British group were aged ≥45 years. The Asian group had the youngest age profile. Higher proportions of the Asian and Mixed/Multiple/Other groups were male; slightly higher proportions were female for all other ethnic groups.

White British people were most likely to be married or cohabiting with a partner. Black people were most likely to be single. Asian and Mixed/Multiple/Other groups were least likely to be divorced or separated.

Black people were the least likely to be owner-occupiers and more likely to be social renters than all other groups. White British people were most likely to be owner-occupiers, followed by Asian people. White Other people were most likely to be private renters compared with other groups.

People in the Asian and White Other groups were most likely to be educated to degree level. Those with a foreign or other qualification were most likely to be White Other. White British people were least likely to have a degree-level education and most likely to have no qualifications.

Around a quarter of people in White British and Black groups, and nearer a third of White Other, Asian, and Mixed/Multiple/Other groups were in a managerial/professional occupation. White British and Asian people were more likely to have never worked or not worked in the past year.

### Univariable analyses

Table 2 presents weighted proportions of prevalence of CMD and different types of treatment for CMD by ethnicity. Prevalence of CMD was highest in Black people, and similar across all other groups (with overlapping confidence intervals). Lower proportions of people from all ethnic minority groups had any form of treatment in the past year relative to White British people.

Almost twice the proportion of White British people as any other ethnic group were taking antidepressants. Black people had the lowest proportion of people who had seen their GP for a mental, emotional or nervous complaint in the past year. White Other people had the lowest proportion, followed by Asian people, but overall proportions were similar – and the same in White British and Black people – for those who had seen a community mental health specialist. Although differences in proportions of people in any counselling or therapy had wide and overlapping confidence intervals, White Other and Asian groups had lower proportions of people in this treatment, where proportions of Black and Mixed/Multiple/Other groups were similar to White British people. Very small proportions of people were currently in counselling or talking therapy, or had seen a community psychiatrist, psychologist or psychiatric nurse in the past year.

### Multivariable analyses

#### CMD

When adjusted for factors of age, gender and survey year, compared with White British people, the odds of CMD prevalence appeared lower in people from White Other and Asian groups, and higher in people from Black and Mixed/Multiple/Other groups; however, these odds ratios had wide and overlapping confidence intervals (model 2, Table 3). When additionally adjusted for marital status, education, tenure and social class, odds remained lower in people from White Other and Asian groups, and were lower for those in the Mixed/Multiple/Other group. Although the odds ratio point estimate was slightly higher in people from the Black group, it moved closer to the null. All these odds ratios also had wide confidence intervals (model 3, Table 3). No evidence of interaction between survey year and prevalence of CMD by ethnicity was found, so it was included as a confounder in this analysis.

Odds of having any CMD were higher in women compared with men; higher in people aged 25–54 years and lower in those aged >65 years, compared with those aged 16–24 years; higher in 2014 compared with 2007; higher in those who were single or separated compared with those who were married or cohabiting with partners; higher in those with no, or GCSE or equivalent qualifications compared with those with a degree-level education; higher in renters compared with owner-occupiers (higher in social renters compared with private renters); and higher in those who had never or not worked in the past year compared with those in managerial or professional occupations, i.e. higher in the most heterogeneous NS-SEC category encompassing some of the most disadvantaged groups, as well as retired people and students, compared with the least disadvantaged group (see Supplementary Table 3 for full results). Barring the foreign/other education category, which encompasses qualifications that could not be classified, weak evidence of a trend of higher odds of CMDs with fewer qualifications was discernible. No trend was seen by social class categories, but differences were evident between least and most disadvantaged groups.

### Any treatment receipt

When adjusted for demographic and socioeconomic factors of age, gender, and testing for interaction with survey year, compared with White British people, odds of treatment receipt appeared lower in all ethnic minority groups in 2007 and 2014 (results are stratified by year, as evidence of interaction was found). However, confidence intervals exceeded 1 for the Mixed/Multiple/Other group across both years, and for all ethnic minority groups in 2007 (model 5, Table 4). After additionally adjusting for marital status, education, tenure, social class, and CMD prevalence, odds for any treatment receipt remained lower in all ethnic minority groups, and effect sizes increased for the Black and Asian groups (model 6, Table 4). In 2007, compared with the White British group, odds were lower for those in the Asian group (odds ratio 0.62, 95% CI 0.39–1.00); confidence intervals exceeded 1 for other ethnic minority groups. In 2014, odds of treatment receipt were lower for the Asian and White Other groups, and lowest in the Black group (odds ratio 0.23, 95% CI 0.13–0.40). Odds were also lower in the Mixed/Multiple/Other group, but confidence intervals exceeded 1.

Adjusted odds of having any treatment were notably higher in those who had any CMD; higher in 2014 compared with 2007; higher in those aged 25–64 years, and lower in those aged >75 years, compared with those aged 16–24 years; higher in those who were separated compared with those who were married or cohabiting with partners; higher in those who were renting compared with owner-occupiers (higher in social renters compared with private renters); and higher in those with no, or GCSE or equivalent qualifications compared with those with a degree-level education (see Supplementary Table 4 for full results). Trends in treatment receipt were not seen by education and social class categories, but only differences between least and most disadvantaged within these groups. There is weak evidence that odds were higher in those with anything below degree-level education and lower in those with foreign/other qualifications, compared with those with a degree-level education, and higher in those who were not working/never worked compared with those in managerial/professional occupations.

## Discussion

Using the latest available data from a nationally representative survey on mental health in England, this study found that overall, differences in CMD prevalence by ethnicity in unadjusted analysis were attenuated after accounting for demographic and socioeconomic factors. However, treatment inequalities persisted for people from ethnic minority backgrounds, with likelihood of treatment receipt lowest in Black people: effect sizes were stronger after accounting for demographic and socioeconomic factors and CMD prevalence.

The evidence of interaction by survey year suggests that the treatment gap has widened over time, in particular for Black people, which is in line with findings from an analysis of APMS data for ethnic inequalities in treatment receipt between 1993 and 2007, where use of antidepressants and GP consultation for mental health problems were lower in people from Black, Asian, Mixed or Other groups compared with the White group.^14^

The effects of the Improving Access to Psychological Therapies (IAPT) programme, launched in 2008 to address inequalities and facilitate access to evidence-based mental health treatments, would not have been reflected in prior findings. The IAPT stepped-care model has a focus on prioritising lower-intensity talking and face-to-face therapies before combining with medication.^15^ However, no evidence of major gains in reducing treatment inequalities was reflected for people from ethnic minority backgrounds in this current analysis for either therapeutic route, in line with wider findings. Evidence from the ethnically diverse London borough of Southwark found that, compared with those referred by GPs, proportions of ethnic minorities who had self-referred to IAPT were more representative of the local population ethnic diversity, indicating that GPs may have been a barrier to accessing IAPT services for these groups.^16^ IAPT referral analysis from Southwark, Lambeth, Croydon and Lewisham demonstrates that, compared with GP referral, racial and ethnic minority groups were more likely to be referred via community services, overall less likely to self-refer to IAPT, and less likely to receive an assessment and then proceed to treatment than White British people.^17^ For those entering IAPT in England between 2018 and 2019, people from the White group were more likely to finish treatment after referral, and reliably improve, than other groups.^2^ These recent findings reflect earlier IAPT demonstration site analysis from the London borough of Newham, where individuals from Black, Asian and Other ethnic minority groups were equally likely to benefit once they gained service access, but were less likely to have attended at least two sessions and concluded involvement with the service; a higher proportion of Black people were represented in the self-referral route.^18^

Increases in treatment receipt between 2007 and 2014 were driven by increased use of psychotropic medication, in particular in White British women.^8,9^ Moreover, it is important to note that White British people are also most likely to be receiving talking therapies; qualitative research with people from ethnic minority backgrounds has found that although medication is seen as a less acceptable form of psychological therapy in these groups, it has been more readily offered, whereas preferences for talking therapies have not been met.^19,20^

Overall, these findings are reflective of the patterns of persisting underrepresentation of people from ethnic minority backgrounds accessing or receiving appropriate mental healthcare from primary care, a longstanding inequality that has been highlighted in previous studies and policy initiatives.^21^ Research to date suggests that these findings of persistent treatment inequalities could be indicative of problems with recognition and diagnosis of CMD in people from ethnic minority backgrounds by service providers, linked to cultural variation in expressions of distress being missed in the consultation process,^22,23^ and discrimination affecting whether treatments are offered equitably across ethnic groups.^16^

Factors behind treatment inequalities may be related to those driving CMD in people from ethnic minority backgrounds, such as intergenerational differences in expression of distress and help-seeking. Variation in cultural and national context may contribute to different experiences and perceptions of mental health problems over the life course. Data from 1993–1994 showed that prevalence of depression was found to be higher in people from Caribbean groups, and lower in those from Asian groups who had recently migrated to the UK, compared with those from White groups; prevalence in those who were 'second generation' was similar.^24^ Further barriers, such as stigma and lack of trust in service providers could be preventing help-seeking for individuals at community level; qualitative research has explored how the effects of cultural norms of self-reliance and resilience, alongside experiences of unfair treatment, and fear and anticipation of coercive treatment, or surveillance by social services, may drive reluctance to engage with mental health services.^19,25^

Previous evidence reviewed above indicates that the relationship between ethnicity and mental health outcomes needs to consider differences by migration history, intergenerational dynamics, gender, ethnic density and area-level factors such as deprivation. Although help-seeking behaviour has not been found to vary by ethnic density, generally lower prevalence of CMDs is observed for people from ethnic minority groups living in areas with higher own-group density.^26^ The link between deprivation and mental health service use should be investigated for people from ethnic minority backgrounds, considering the close relationship between socioeconomic and ethnic health inequalities in the UK.^27^ Further analysis of these mechanisms resulting in divergent experiences of, and pathways to care for, CMDs could help to explain factors driving these persisting disparities. Clinical implications of this research include improving accessibility of existing psychological therapy provision for CMDs, and designing mental health services appropriate for diverse and marginalised population groups where needs are not being met by existing provision.

### Strengths and limitations

This is the first analysis of ethnicity, CMDs and mental health treatments in a large and nationally representative general population sample since the Ethnic Minority Psychiatric Illness Rates in the Community (EMPIRIC) survey more than two decades ago.^4^ EMPIRIC sampling did not include people from Black African backgrounds, a significant population who are included here, albeit within an aggregated Black group. A key limitation of this analysis was the smaller sample size of ethnic minority groups, which may not have been adequately powered to detect small differences between diverse ethnicities disaggregated by gender (with only a binary variable for gender available for analysis here). A systematic review of mental health surveys between 1999 and 2010 in the UK found that sampling strategies were often not designed or resourced to recruit adequate numbers of ethnic minority participants.^5^

Although ethnicity was self-identified and it was possible to distinguish between White British and White Other in this study, heterogeneity was still obscured in comparing only four ethnic minority groups, in people who were not distinguishable by migration status. There are too many ethnic groups represented in the Mixed/Multiple/Other ethnic category to form any meaningful conclusions regarding any underlying mechanisms in the relationship between ethnicity and CMD.

Any effects of racism and discrimination, which have been documented to affect mental health and well-being for ethnic minority groups, were not explored here.^28^ Lack of sympathy or even antagonism to disclosure of racism in therapeutic encounters has been cited as barriers to accessing care, alongside the Eurocentric nature of psychological therapies.^19^ In a forthcoming analysis of APMS 2014 data, experiences of discrimination masked a lower likelihood of mental health service use in Black people after adjustment for symptoms, and was associated with greater treatment use in those who identified as gay, lesbian, bisexual or other sexuality.^29^

More limited help-seeking in ethnic minority groups, related to lack of trust and anticipated discrimination from service providers, has also been explored previously. Stigma regarding mental illness, differing explanatory models of mental illness and approaches to help-seeking for mental health, doubts about cultural competence of service providers, the dynamics of informal help-seeking, and alternative therapies have been explored as reasons behind differing mental health treatment use in ethnic minority groups, and are important questions for further enquiry, alongside experiences and access to mainstream services.^19,30^

APMS interviews were only conducted in English, so a key survey limitation is participation issues related to the language barrier experienced by people from ethnic minority backgrounds with limited English. Further sampling issues affecting this analysis include the cross-sectional nature of the APMS data, and sampling design limitations meaning only those living in private households are included here, rather than people living in institutions, who may face increased vulnerabilities affecting mental health. Large proportions of eligible participants do not respond to population health surveys, although APMS response rates are in line with similar surveys.^8^ Methodological limitations in this analysis using multi-variable regression also include the complex relationship between socioeconomic status, ethnicity and mental health, as socioeconomic confounders potentially lie on the causal pathway between ethnicity and the outcomes (CMD prevalence and mental health treatment receipt). The two-stage adjustment presented here was applied to demonstrate the effect of adjusting for demographic factors separately from factors indicating socioeconomic position.

Although the psychosocial impact of the COVID-19 pandemic is yet to be felt and assessed comprehensively, emerging evidence suggests increased mental health deterioration for people from ethnic minority groups in the UK, who have been disproportionately affected.^31^ Economic loss, health issues and social isolation experienced in the period of lockdown associated with pandemic response measures may have short- to long-term effects. As models of mental health help-seeking, treatment, referral and service delivery are also undergoing change in this transitional time, caution is required so that existing inequalities are not replicated in future.

In conclusion, although no significant changes in prevalence of CMDs have been found over time, persisting, if not widening, CMD treatment inequalities continue, which are not explained by known demographic and socioeconomic confounders, particularly for Black people. Demographic and socioeconomicfactors obscured differences in mental health treatment access by ethnicity, which emerged in adjusted analysis. Adequately powered surveys that are designed to examine the intersections of ethnic identity, gender, age, socioeconomic inequalities, migration and generational status with respect to mental health outcomes are urgently required, to address the limitations of existing studies and better understand persistent inequalities.

## Figures and Tables

**Table 1:** Demographic and socioeconomic overview of 2007 and 2014 Adult Psychiatric Morbidity Survey complete-case data combined

Factor	White British	White Other	Black	Asian	Mixed/Multiple/Other	Whole sample
Combined sample	12653 (86.7)	718 (4.9)	375 (2.6)	547 (3.7)	307 (2.1)	14 600 (100)
Gender						
Male	5278 (48.5)	275 (46.0)	143 (46.1)	257 (56.1)	128 (51.3)	6081 (48.8)
Female	7375 (51.5)	443 (54.0)	232 (53.9)	290 (43.9)	179 (48.7)	8519 (51.2)
						3.2, *P* = 0.0141
Age, years						
16–24	866 (12.8)	63 (14.5)	35 (16.8)	87 (26.0)	46 (21.5)	1097 (14.0)
25–34	1558 (14.8)	188 (32.0)	81 (23.8)	139 (26.6)	70 (25.8)	2036 (17.0)
35–44	2049 (15.1)	161 (20.2)	96 (22.4)	164 (23.5)	83 (22.4)	2553 (16.3)
45–54	2079 (18.1)	93 (12.3)	79 (20.6)	67 (12.0)	57 (16.8)	2375 (17.4)
55–64	2232 (15.0)	85 (9.3)	38 (7.5)	42 (5.6)	34 (9.6)	2431 (13.8)
65–74	2015 (13.1)	72 (6.9)	27 (5.1)	33 (4.3)	11 (2.6)	2158 (11.7)
75+	1854 (11.2)	56 (4.7)	19 (3.8)	15 (2.0)	6 (1.2)	1950 (9.9)
						21.7, *P* < 0.0001
Marital status						
Single	2374 (21.2)	174 (28.4)	161 (43.0)	138 (36.3)	96 (32.7)	2943 (23.4)
Married/cohabitating	7067 (63.6)	407 (60.8)	131 (41.5)	324 (55.6)	167 (58.6)	8096 (62.2)
Divorced/separated	3212 (15.2)	137 (10.8)	83 (15.6)	85 (8.1)	44 (8.6)	3561 (14.4)
						21.7, *P* < 0.0001
Home tenure						
Owner-occupier	8955 (71.1)	364 (45.8)	140 (37.5)	334 (59.7)	151 (42.5)	9944 (67.2)
Private renter	1542 (13.5)	241 (40.5)	72 (22.3)	136 (25.4)	87 (33.3)	2078 (16.6)
Social renter	2156 (15.3)	113 (13.7)	163 (40.3)	77 (14.9)	69 (24.2)	2578 (16.2)
						58.8, *P* < 0.0001
Education						
Degree	2484 (20.4)	261 (37.7)	93 (26.0)	213 (38.2)	93 (30.6)	3144 (22.8)
Teaching, nursing, HND	986 (7.6)	50 (6.7)	47 (11.7)	24 (4.1)	35 (9.1)	1142 (7.5)
A level	1876 (17.2)	77 (10.9)	54 (15.1)	81 (19.0)	35 (12.0)	2123 (16.8)
GCSE/equivalent	3200 (27.2)	94 (15.0)	78 (22.1)	93 (18.4)	70 (26.5)	3535 (25.9)
Foreign/other	417 (2.7)	67 (9.5)	17 (3.8)	31 (4.4)	23 (6.3)	555 (3.3)
No qualifications	3690 (24.8)	169 (20.3)	86 (21.4)	105 (15.9)	51 (15.5)	4101 (23.7)
						14.6, *P* < 0.0001
Social class (NS-SEC)						
Managerial/professional	3016 (25.9)	204 (29.4)	104 (26.4)	172 (31.2)	89 (28.0)	3585 (26.5)
Intermediate	987 (8.1)	61 (7.8)	33 (8.0)	23 (4.6)	28 (8.5)	1132 (7.9)
Small employers/own account workers	697 (5.9)	49 (6.8)	19 (5.6)	29 (5.7)	18 (5.6)	812 (6.0)
Lower supervisory/ technical occupations	480 (4.5)	26 (4.4)	14 (3.9)	24 (4.5)	13 (3.2)	557 (4.5)
Semi-routine/routine occupations	1895 (17.6)	127 (21.0)	85 (25.8)	88 (16.7)	67 (23.7)	2262 (18.2)
Never worked/not worked in past year	5578 (37.9)	251 (30.6)	120 (30.3)	211 (37.3)	92 (31.0)	6252 (37.0)
						2.1, *P* < 0.0001
Survey year						
2007	6351 (50.3)	302 (43.0)	181 (48.2)	195 (35.0)	158 (51.9)	7187 (49.1)
2014	6302 (49.7)	416 (57.0)	194 (51.8)	352 (65.0)	149 (48.1)	7413 (50.9)
						10.0, *P* < 0.0001

Data are shown as *n* (weighted proportions %), with Pearson’s *χ*^2^-test adjusted design-based *F*-values and *P*-values. HND, Higher National Diploma; NS-SEC, National Statistics Socio-economic Classification.

**Table 2:** Distribution of mental health outcomes in Adult Psychiatric Morbidity Survey 2007 and 2014 data, by ethnicity (total sample *N* = 14 600)

	Weighted proportion % (95% confidence interval), (*n*)					
Ethnicity	Prevalence of CMDs (CIS-R > 12)	Seen GP for a mental, nervous or emotional complaint	Seen community mental health specialist	In any counselling/therapy	Taking antidepressants	Received any of these treatments for a CMD
White British (*n* = 12 653)	16.2 (15.5–17.0) (2158)	12.2 (11.6–12.8) (1694)	2.1 (1.8–2.4) (276)	2.7 (2.4–3.1) (376)	7.9 (7.4–8.4) (1144)	16.1 (15.4–16.8) (2246)
White Other (*n* = 718)	15.4 (12.7–18.6) (123)	8.9 (7.0–11.4) (84)	1.6 (0.8–3.0) (14)	2.0 (1.1–3.5) (17)	4.6 (3.3–6.5) (47)	12.5 (10.0–15.5) (111)
Black (*n* = 375)	22.7 (17.8–28.5) (86)	8.2 (5.6–11.9) (39)	2.1 (1.1–4.1) (10)	2.8 (1.5–5.3) (11)	3.4 (1.8–6.3) (12)	11.3 (8.1–15.5) (50)
Asian (*n* = 547)	16.3 (13.3–19.8) (98)	9.3 (7.1–12.2) (60)	1.9 (0.8–4.3) (7)	2.2 (1.0–4.6) (11)	3.0 (1.8–4.9) (20)	11.9 (9.5–14.8) (71)
Mixed/Multiple/Other (*n* = 307)	19.3 (14.9–24.7) (70)	11.6 (8.4–15.7) (45)	1.9 (0.9–4.1) (8)	2.9 (1.4–6.0) (9)	2.0 (1.0–3.9) (11)	13.8 (10.4–18.2) (53)
Whole sample (*N* = 14600)	16.4 (15.7–17.2) (2535)	11.7 (11.1–12.3) (1922)	2.0 (1.8–2.3) (315)	2.7 (2.4–3.0) (424)	7.1 (6.7–7.6) (1234)	15.5 (14.8–16.1) (2531)
Pearson’s *χ*^2^-test^a^	*F* = 2.5, *P* = 0.04	*F* =3.3, *P* = 0.011	*F* =0.2, *P* = 0.94	*F* =0.4, *P* = 0.80	*F* =11.8, *P* <0.0001	*F* =4.3, *P* = 0.0020

CMD, common mental disorder; CIS-R, Clinical Interview Schedule – Revised.

a Data shows Pearson’s *χ*^2^-test adjusted design-based *F*-value, *P*-value.

**Table 3:** Logistic regression model results for prevalence of common mental disorders (CIS-R > 12) in Adult Psychiatric Morbidity Survey 2007 and 2014 data (*N* = 14 600)

Ethnicity	Unadjusted (model 1)	Adjusted for age, gender and survey year (model 2)	Adjusted for age, gender, marital status, education, tenure, social class, and survey year (model 3)
White British	1.00 (Reference)	1.00 (Reference)	1.00 (Reference)
White Other	0.94 (0.75–1.19)	0.85 (0.67–1.08)	0.83 (0.64–1.06)
Black	1.52 (1.11–2.08)	1.35 (0.98–1.86)	1.04 (0.75–1.44)
Asian	1.00 (0.79–1.28)	0.92 (0.71–1.17)	0.86 (0.66–1.12)
Mixed/Multiple/Other	1.24 (0.90–1.70)	1.11 (0.80–1.52)	0.96 (0.69–1.35)
Adjusted Wald test results for association of ethnicity with outcome	*F* = 2.2, *P* = 0.07	*F* = 1.8, *P* = 0.14	*F* = 0.9, *P* = 0.47

All data are shown as odds ratios (95% confidence intervals) unless otherwise stated. Model 2: no evidence of ethnicity×survey year interaction found: *F* = 1.28, *P* = 0.24. Model 3: no evidence of ethnicity×survey year interaction found: *F* = 1.02, *P* = 0.42. CIS-R, Clinical Interview Schedule – Revised.

**Table 4:** Logistic regression model results for any treatment receipt for a common mental disorder in Adult Psychiatric Morbidity Survey 2007 and 2014 data (*N* = 14 600)

	Unadjusted (model 4) Combined 2007 and 2014 sample	Adjusted for age, gender and ethnicity×survey year interaction (model 5)		Adjusted for age, gender, marital status, education, tenure, social class, CMD prevalence and ethnicity×survey year interaction (model 6)	
Ethnicity		2007	2014	2007	2014
White British	1.00 (Reference)	1.00 (Reference)	1.00 (Reference)	1.00 (Reference)	1.00 (Reference)
White Other	0.74 (0.57–0.96)	0.85 (0.59–1.22)	0.57 (0.39–0.82)	0.78 (0.51–1.18)	0.58 (0.38–0.87)
Black	0.66 (0.46–0.96)	0.90 (0.53–1.54)	0.40 (0.24–0.68)	0.68 (0.38–1.23)	0.23 (0.13–0.40)
Asian	0.71 (0.55–0.91)	0.69 (0.46–1.02)	0.65 (0.47–0.90)	0.62 (0.39–1.00)	0.60 (0.42–0.85)
Mixed/Multiple/Other	0.84 (0.60–1.16)	0.76 (0.47–1.24)	0.82 (0.53–1.25)	0.65 (0.39–1.11)	0.67 (0.39–1.14)
Adjusted Wald test results for association of ethnicity with outcome	*F* =4.1, *P* = 0.003	*F* =1.4, *P* = 0.22		*F* = 2.2, *P* = 0.07	

All data are shown as odds ratios (95% confidence intervals) unless otherwise stated. Model 5: ethnicity×survey year interaction: *F* = 8.9, *P* < 0.0001. Model 6: ethnicity×survey year interaction: *F* = 10.60, *P* < 0.0001. CMD, common mental disorder.

## Data Availability

APMS data is deposited with the UK Data Service at the University of Essex. An end-user licence was agreed to access the 2007 data. APMS data from 2014 was obtained via special licence through a data-sharing agreement with NHS Digital Data Access Request Service.
